# Systemic Inflammation Differences in Brain-vs. Circulatory-Dead Donors: Impact on Lung Transplant Recipients

**DOI:** 10.3389/ti.2024.12512

**Published:** 2024-06-03

**Authors:** Alberto Sandiumenge, Irene Bello, Elisabeth Coll-Torres, Aroa Gomez-Brey, Clara Franco-Jarava, Eduardo Miñambres, Marina Pérez-Redondo, Fernando Mosteiro, Laura Sánchez-Moreno, Silvana Crowley, Eva Fieira, Borja Suberviola, Cristopher Alan Mazo, Alvar Agustí, Teresa Pont

**Affiliations:** ^1^ Donation and Trasplantation Program Coordination Unit, Vall d’Hebron, University Hospital, Cell, Tissue and Organ Donation and Trasplantation Resarch Group, Vall d’Hebron Research Institute (VHIR), Barcelona, Spain; ^2^ Department of Thoracic Surgery, Respiratory Institute Hospital Clínic, Barcelona, Spain; ^3^ Institut d’Investigacions BIomediques August Pi i Sunyer (IDIBAPS), Barcelona, Spain; ^4^ Medical Department, Organización Nacional de Trasplantes, Madrid, Spain; ^5^ Immunology Department, Vall d’Hebron University Hospital, Barcelona, Spain; ^6^ Transplant Coordination Unit and Department of Intensive Care, University Hospital Marqués de Valdecilla-IDIVAL, School of Medicine, University of Cantabria, Santander, Spain; ^7^ Departament of Critical Care, Department of Donor and Transplant Coordinator, Puerta de Hierro Majadahonda University Hospital, Madrid, Spain; ^8^ Departament of Critical Care, Department of Donor and Transplant Coordinator, A Coruña University Hospital, A Coruña, Spain; ^9^ Department of Thoracic Surgery, University Hospital Marqués de Valdecilla-IDIVAL, School of Medicine, University of Cantabria, Santander, Spain; ^10^ Department of Thoracic Surgery and Lung Trasplantation, Puerta de Hierro Majadahonda University Hospital, Madrid, Spain; ^11^ Department of Thoracic Surgery and Lung Trasplantation, A Coruña University Hospital, A Coruña, Spain; ^12^ Department of Pneumology, Respiratory Institute, Barcelona, Spain; ^13^ Cátedra de Salut Respiratoria, Universidad de Barcelona, Barcelona, Spain; ^14^ CIBER Enfermedades Respiratorias (CIBERES), Madrid, Spain

**Keywords:** lung transplantation, brain-death donation, circulatory-death donation, interleukin, cytokine storm

## Abstract

Brain death triggers a systemic inflammatory response. Whether systemic inflammation is different in lung donors after brain- (DBD) or circulatory-death (DCD) is unknown, but this may potentially increase the incidence of primary graft dysfunction (PGD) after lung transplantation. We compared the plasma levels of interleukin (IL)-6, IL-8, IL-10 and TNF-α in BDB and DCD and their respective recipients, as well as their relationship with PGD and mortality after LT. A prospective, observational, multicenter, comparative, cohort-nested study that included 40 DBD and 40 DCD lung donors matched and their respective recipients. Relevant clinical information and blood samples were collected before/during lung retrieval in donors and before/during/after (24, 48 and 72 h) LT in recipients. Incidence of PGD and short-term mortality after LT was recorded. Plasma levels of all determined cytokines were numerically higher in DBD than in DCD donors and reached statistical significance for IL-6, IL-10 and IL-8. In recipients with PGD the donor’s plasma levels of TNF-α were higher. The post-operative mortality rate was very low and similar in both groups. DBD is associated with higher systemic inflammation than DCD donors, and higher TNF-α plasma levels in donors are associated with a higher incidence of PGD.

## Graphical Abstract

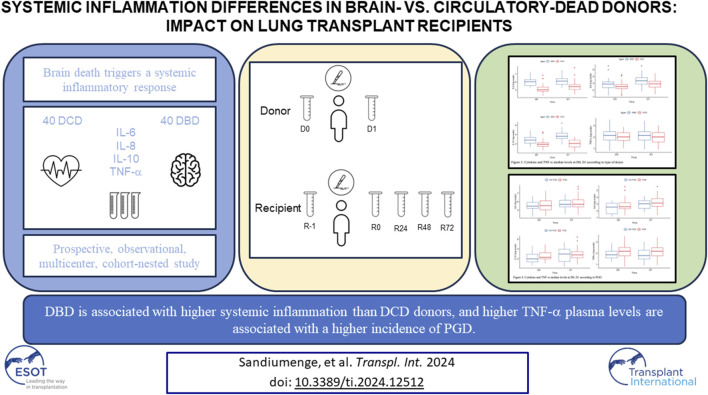

## Introduction

Donation after brain death (DBD) is the main source of organ donation for transplantation worldwide. Brain death (BD) usually induces a systemic inflammatory response. This “cytokine storm” may damage different body organs in donors, which may in turn have a deleterious impact on their function and survival in recipients after transplantation [1–3] since this can further aggravate the insults that occur during warm and cold ischemia and the subsequent reperfusion of the transplanted organ, by amplifying an inflammatory response in the recipient [4]. Lungs are especially sensitive to the BD-induced cytokine storm, which enhances the likelihood of ischemia-reperfusion-induced primary graft dysfunction (PDG) [5]. PDG is one of the main complication during the early post-operative period of lung transplantation and is the first cause of mortality during the first month and second one during the first year after transplantation [6] significant morbidity, as well as longer hospital length of stay and duration of mechanical ventilation. Experimental evidence has strongly suggested that DBD increases the incidence and severity of PGD(3,7).

Given the shortage of DBD donors, in recent years, donation after circulatory death (DCD) has been increasingly used as a source of organs for transplantation. The cytokine storm that follows BD and the potential deleterious impact on the lungs could theoretically be prevented or minimized during a DCD process [7]. Based on previous studies, we hypothesized that the plasma level of several inflammatory cytokines would be higher in DBD vs. DCD. To test this hypothesis, we conducted a prospective study that sought to compare: (1) the plasma levels of the pro-inflammatory (IL-6, IL-8, TNF-α) and anti-inflammatory cytokines (IL-10) in BDD vs. DCD; (2) the plasma levels of pro-inflammatory (IL-6, IL-8, TNF-α) and anti-inflammatory cytokines (IL-10) in recipients with PGD; and (3) the incidence of PGD and short-term mortality in LT recipients from DBD or DCD.

## Material an Methods

### Study Design and Ethics

This was a prospective, observational cohort-nested study conducted in four transplant centers in Spain that included adult patients undergoing uni- or bilateral LT between July 2018 and July 2019 and their respective BD or CD donors. The type of death was certified in accordance with Spanish legislation^25^.

The study protocol conformed to the ethical guidelines of the 1975 Declaration of Helsinki as reflected in *a priori* approval by the Human Research Committees of all participating hospitals (PR [AG]202/2017). All recipients provided written informed consent before being included in the study. Informed consent to participate in the study from donors was included in the donation consent. No deviation from the standard management of lung recipients took place except for serial blood sampling.

### Patients

**FIGURE 1:**
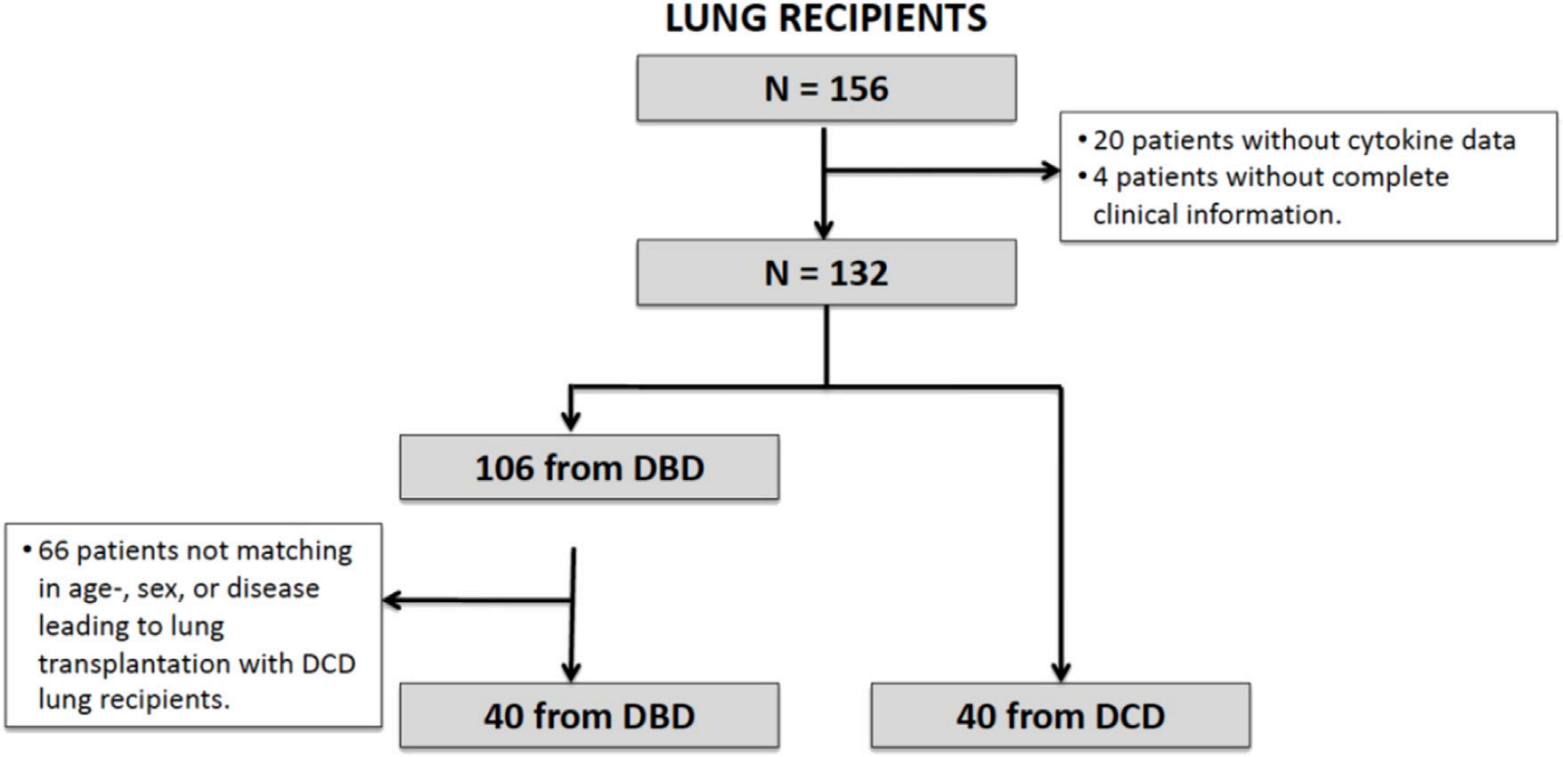
Consort diagram of the study.

LT recipients from DBD and DCD were matched individually for sex, age (±5 years), and indication for LT (Figure 1). We excluded from this analysis patients undergoing lung re-transplantation, cardiopulmonary transplantation or those who had previously undergone or were to receive more than one simultaneous solid organ transplantation, as well as those for whom the graft ischemic time was >420 min. Following previous reports by Koukoulis et al.^27^ and considering a confidence interval of 95%, statistical power of 80% and patient loss of 15%, a sample size of 36 LT recipients for each group (DCD and DBD) was estimated to detect a difference of at least one SD (15 pg/mL) in IL-6 plasma levels.

### Study Variables

The following donor and recipient variables were prospectively collected: demographic (sex, age) and anthropometric (body mass index) measurements; clinical data (cause of death, corticosteroid pretreatment of donors and indication for transplantation of recipients); and surgical data (transfusion of blood products, vasoactive support or application of veno-venous [VV-] or veno-arterial [VA-] extracorporeal membrane oxygenation [ECMO], ischemic times for the first and second lungs and use of cardiopulmonary bypass). In all recipients, we registered the development of PGD (any grade and grade III) within 72 h post-transplantation according to the International Society for Heart and Lung Transplantation (ISHLT) Working Group criteria [8]. Mortality during the first 3 months after the transplant was also recorded.

### Measurements

Blood samples (10 mL) were obtained from donors before skin incision (D0) and before organ perfusion (D1). In recipients, blood samples were obtained in the operating room before implantation surgery (R-1), just after graft reperfusion (R0) and 24 (R24), 48 (R48) and 72 (R72) hours after LT. All blood samples were collected in EDTA tubes and centrifuged at 1,000 g for 10 min at room temperature (22ºC–23°C). Plasma was separated, divided into 2 aliquots of 2 mL each, and immediately stored at −80°C until analysis. At the end of the inclusion period, samples were shipped together in dry ice containers to the central laboratory located at the coordinating site (Hospital Universitari Vall d’Hebron, Barcelona) for cytokine analysis by immunofluorescence assays based on microfluidics using ELLA Simple Plex (Protein Simple, Biotechne, CA, United States) to simultaneously detect IL-6, IL-10, IL-8 and TNF-α. Triplicates of each cytokine result were obtained (the maximum allowed variation among triplicates was 5%).

### Data Analysis

Data were collected and stored in an *ad hoc* database on the website of the *Organización Nacional de Trasplantes* (ONT, Spanish Transplant Organization) and made accessible only to the principal investigator of each participating site. A member of the ONT was commissioned to monitor the study.

The Shapiro-Wilk test was performed as a test of normality for IL level distribution. Categorical variables are expressed as n and percentages. Quantitative data is presented as mean ± SD if normally distributed or as median (Q1, Q3) if not. Chi-square test, or Fisher’s exact test were used to compare categorical variables. Change over time was analyzed with non-parametric 2-way mixed repeated-measures ANOVA. The basic model included a group factor (DBD or DCD), a time factor, and an interaction between group and time. The main effects of group and time were explored for nonsignificant interactions. Bonferroni correction was applied to adjust for multiple comparisons in posthoc tests to determine specific pairwise differences between groups. All statistical analyses were performed using R version 4.3.1 (R Core Team, 2023). A *p*-value < 0.05 was considered statistically significant.

## Results

### Patient Characteristics

Figure 1 presents the consort diagram of the study. A total of 156 LT recipients were initially included, but 24 were later excluded because of incomplete data. Of the remaining 132 patients, 106 were DBD recipients and 40 were DCD recipients. Forty of the 106 DBD recipients were matched individually to the 40 DCD ones by sex, age (±5 years) and indication for lung transplantation.

Table 1 contrasts the main characteristics of both donors and recipients. More DBD received corticosteroids and vasoactive support before lung retrieval than DCD (*p* = 0.02 and *p* = 0.043, respectively), while more DCD than DBD received transfusions of blood products (*p* = 0.001). No other significant differences were observed between groups in donor or recipient characteristics.

**TABLE 1:** Donor and recipient characteristics.

		DBD	DCD	*p* -value
		(n = 40)	(n = 40)	
Donor	Sex, male, n (%)	22 (55)	18 (45)	0.799
	Age, years, mean ± SD	54 ± 16	56 ± 14	0.435
	BMI, kg/m^2^, mean ± SD	26 ± 5	26 ± 5	0.754
	Corticosteroids treatment, n (%)	35 (87.5)	22 (55)	0.002
	Transfusion with blood products, n (%)	2 (5)	8 (20)	0.043
	Vasoactive drugs treatment, n (%)	34 (85)	12 (30)	0.001
	VV- or VA-ECMO, n (%)	0 (0)	4 (10)	0.116
	Cause of death, n (%)			0.014
	Stroke	31 (77.5)	19 (47.5)	
	Anoxia	3 (7.5)	13 (32.5)	
	Traffic head injury	2 (5)	1 (2.5)	
	Traffic non head injury	4 (10)	4 (10)	
	Other	0 (0)	3 (7.5)	
Recipient	Sex, male, n (%)	23 (57.5)	24 (60)	0.820
	Age, years, mean ± SD	56 ± 10	54 ± 10	0.500
	BMI kg/m^2^± SD	25 ± 4	24 ± 4	0.189
	Ischemic time 1st graft, min, mean ± SD	238 ± 61	256 ± 48	0.213
	Ischemic time 2nd graft, min, mean ± SD	341 ± 82	353 ± 56	0.213
	Cardiopulmonary bypass, n (%)	20 (20)	7 (17.5)	0.775
	Transfusion with blood products, n (%)	27 (67.5)	19 (47.5)	0.070
	Vasoactive drugs intake, n (%)	33 (82.5)	35 (87.5)	0.630
	VV- or VA-ECMO after transplantation, n (%)	2 (5)	4 (10)	0.675
	Indication for lung transplantation (%)			-
	Bronchiectasis	3 (7.5)	3 (7.5)	
	Diffuse interstitial lung disease	17 (42.5)	18 (45)	
	Occupational lung disease	1 (2.5)	1 (2.5)	
	COPD/Emphysema	12 (30)	11 (27.5)	
	Cystic fibrosis	3 (7.5)	3 (7.5)	
	Pulmonary hypertension	3 (7.5)	3 (7.5)	
	Other	1 (2.5)	1 (2.5)	

BMI: body mass index; COPD, chronic obstructive pulmonary disease; ECMO: extracorporeal membrane oxygenation; VA: veno-arterial; VV: veno-venous.

### Systemic Inflammation

**FIGURE 2:**
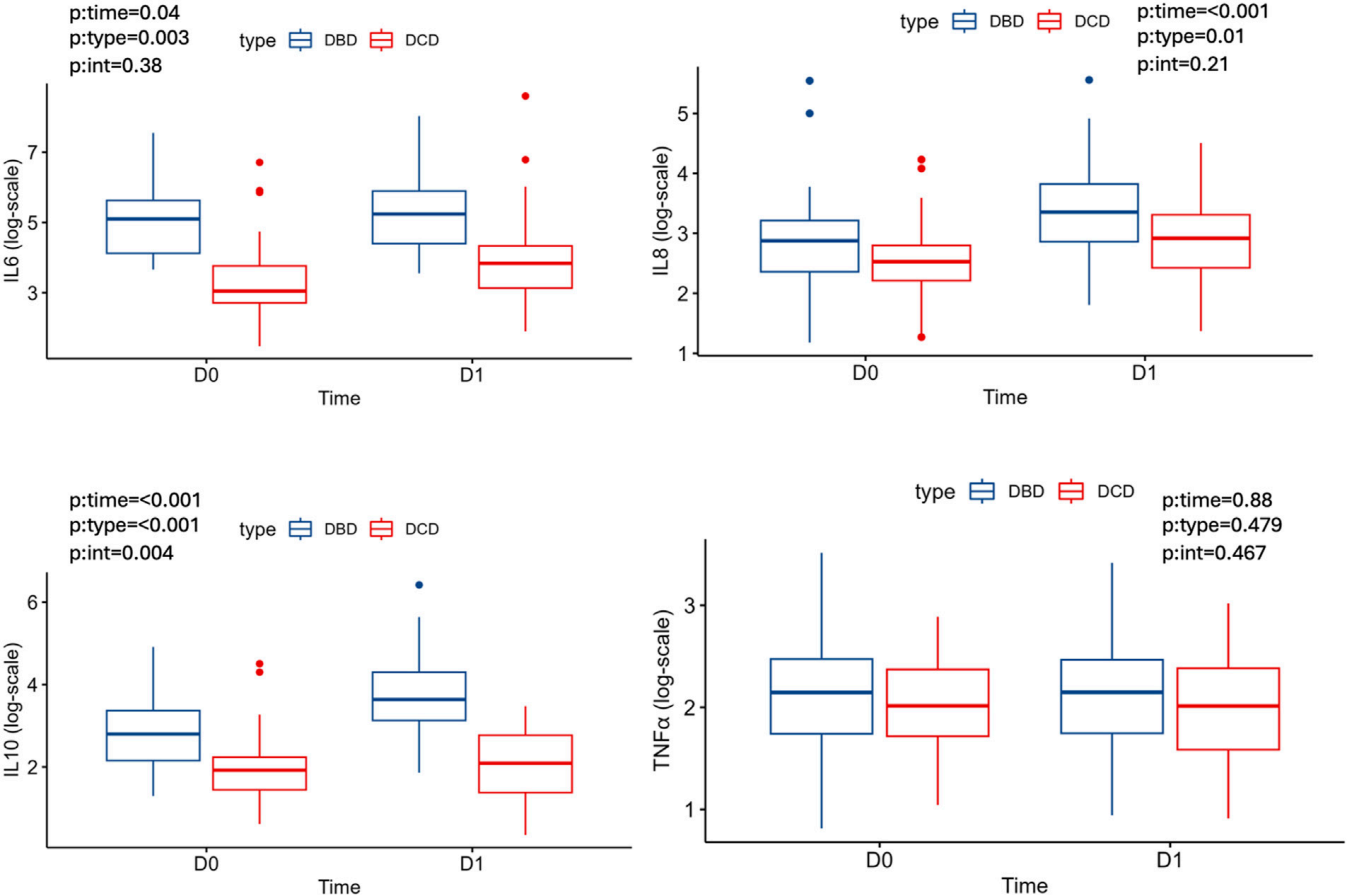
Cytokine and TNF-α median levels at D0, D1 according to type of donor.

At D0 and D1 the levels of IL-6, IL-10 and IL-8 were higher in the DBD group than in DCD, and a significant main effect of time with a higher concentrations at D1 compared to D0 with statistical significant increase the IL-10 levels at D1 in DBD group. There was no statistical differences of type or time on TNFα (Table 2; Figure 2).

**TABLE 2:** Levels (pg/mL) of cytokines according to the type of donor at D0 and D1. Data expressed as median (Q1, Q3). *p*-values corresponding to non-parametric two-way mixed ANOVA.

	D0		D1		*p*-values		
	DBD	DCD	DBD	DCD	p:time	p:type	p:interaction
IL10	16.4 (8.6, 29.0)	6.8 (4.2, 9.4)	37.9 (22.8, 73.7)	8.1 (4.0, 15.9)	<0.001	<0.001	0.004
IL 6	164.3 (61.9, 278.9)	21.1 (15.1, 43.2)	189.4 (81.5, 363.3)	46.5 (23.0, 76.3)	0.040	0.003	0.381
IL 8	18.1 (11.3, 25.3)	12.5 (9.1, 16.5)	28.6 (17.5, 45.7)	18.5 (11.3, 27.4)	<0.001	0.010	0.210
TNF α	8.6 (5.7, 11.9)	7.5 (5.6, 10.7)	8.6 (5.7, 11.8)	7.5 (4.9, 10.8)	0.883	0.479	0.467

**FIGURE 3:**
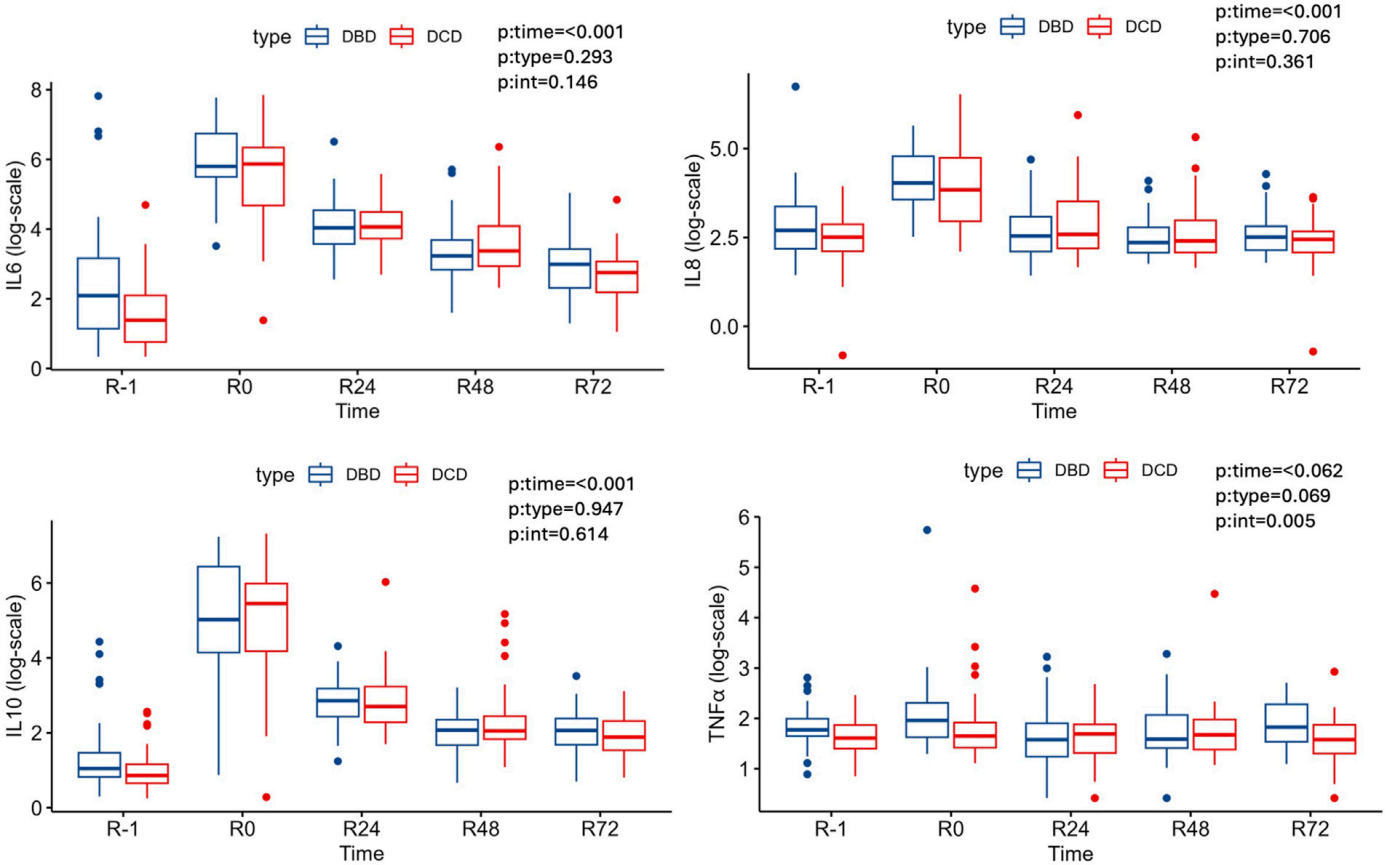
Cytokine and TNF-α median levels at R-1, R0, R24, R48, R72 according to type of donor.

In recipients, there was no statistical significance of type of donor on IL-6, IL-10, IL-8 and TNF. Before LT (R-1), the plasma level of these cytokines was similar in both groups (DBD and DCD) and immediately after LT (R0), the plasma levels of IL-6, IL-8 and IL-10, increased in both groups of recipients and decreased thereafter during the next 72 h without differeces. There was a statistically significant effect of time on TNFα only for the DBD group (Table 3; Figure 3).

**TABLE 3:** Levels (pg/mL) of cytokines according to the type of donor at R-1 to R72. Data expressed as median (Q1, Q3). *p*-values corresponding to non-parametric two-way mixed ANOVA.

	R-1		R0		R24		R48		R72		*p*-values		
	DBD	DCD	DBD	DCD	DBD	DCD	DBD	DCD	DBD	DCD	p:time	p:type	p:interaction
IL10	2.9 (2.3, 4.3)	2.4 (1.9, 3.2)	151.6 (63.7, 624.0)	233.3 (69.2, 397.4)	17.4 (11.4, 24.1)	14.9 (9.8, 25.4)	7.9 (5.3, 10.5)	7.8 (6.3, 11.5)	7.9 (5.4, 10.8)	6.6 (4.6, 10.1)	<0.001	0.947	0.614
IL 6	8.1 (3.1, 23.7)	4.0 (2.1, 8.1)	329.2 (244.3, 848.8)	352.5 (107.4, 565.9)	56.7 (35.7, 93.4)	58.0 (41.6, 89.1)	25.3 (17.1, 39.9)	29.2 (18.9, 59.5)	19.9 (10.1, 30.7)	15.7 (8.9, 21.6)	<0.001	0.293	0.146
IL 8	14.8 (8.8, 29.0)	12.3 (8.2, 17.6)	56.2 (35.2, 119.0)	46.7 (19.1, 114.7)	12.7 (8.2, 21.8)	13.3 (9.0, 33.5)	10.6 (7.9, 16.2)	11.0 (8.0, 19.8)	12.3 (8.5, 16.7)	11.5 (8.0, 14.4)	<0.001	0.706	0.361
TNF α	5.9 (5.2, 7.3)	5.0 (4.1, 6.5)	7.1 (5.1, 10.1)	5.2 (4.1, 6.8)	4.8 (3.5, 6.7)	5.4 (3.7, 6.6)	4.9 (4.1, 7.9)	5.3 (4.0, 7.2)	6.2 (4.7, 9.8)	4.9 (3.7, 6.5)	0.062	0.069	0.005

### Primary Graft Dysfunction (PGD)

**FIGURE 4:**
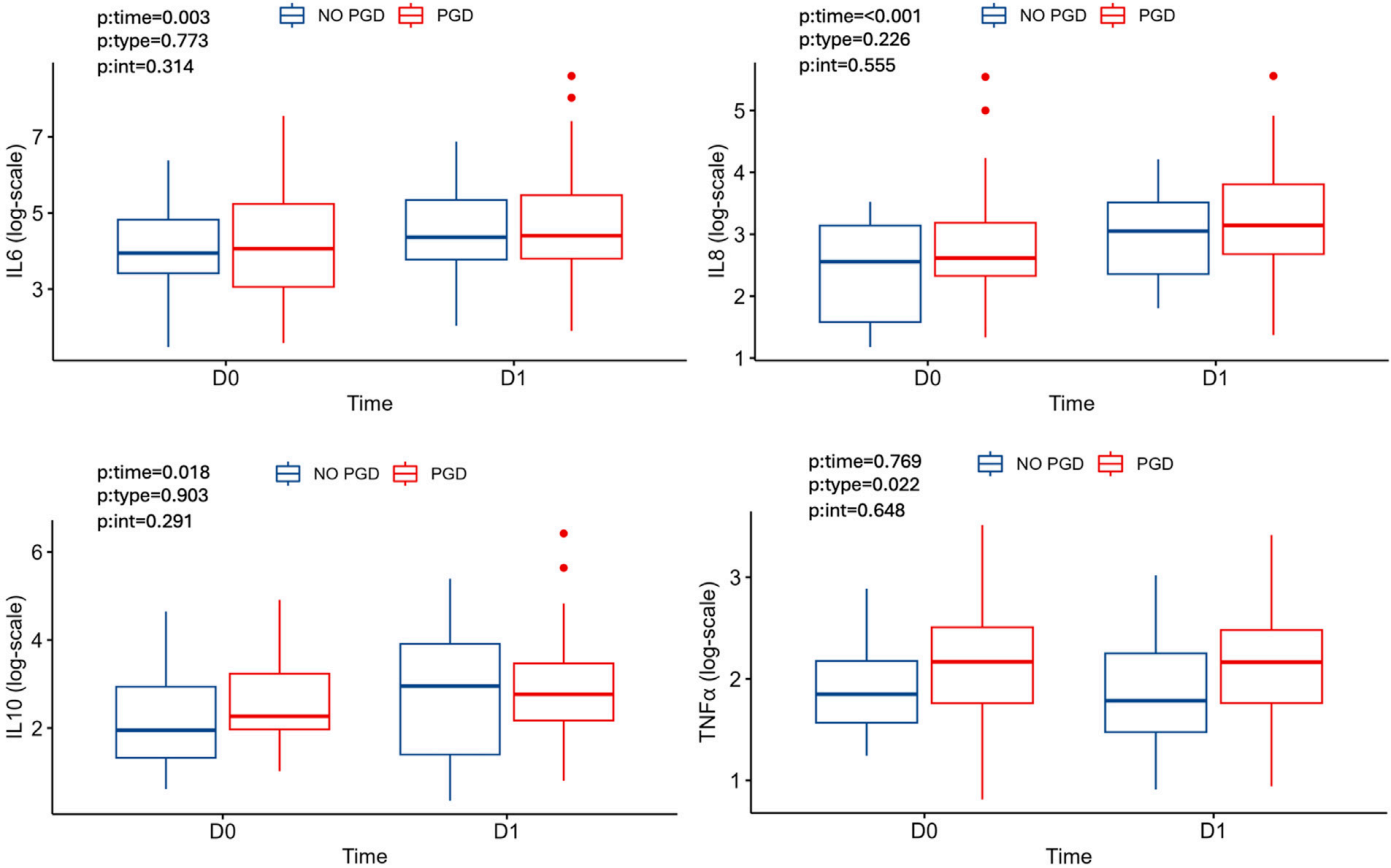
Cytokine and TNF-α median levels at D0, D1 according to PGD.

The incidence of PGD of any grade was similar in DCD and DBD recipients (Table 4). Therefore, to further investigate the evolution of systemic inflammation in PGD we merged both groups for analysis. We found that the donor plasma levels of all cytokines were comparable between patients who developed any grade of PGD or not, except for TNF-α, which was higher at D0 and D1 in those recipients with PGD (Table 5; Figure 4). Specific analysis of PGD grade 3 did not show differences between groups (Table 6).

**TABLE 4:** Outcomes after lung transplantation from DBD and DCD.

	DBD	DCD	*p* -value
	(n = 40)	(n = 40)	
Primary graft dysfunction, n (%)			
Of any grade	27 (67.5)	26 (65)	0.100
Grade III	14 (35)	11 (27.5)	0.469
Post-operative mortality	0 (0)	2 (5)	0.494
Three-month mortality	0 (0)	2 (5)	0.494

**TABLE 5:** Levels (pg/mL) of cytokines according to PGD at D0 and D1. Data expressed as median (Q1, Q3). *p*-values corresponding to non-parametric two-way mixed ANOVA.

	D0		D1		*p*-values		
	No PGD	PGD	No PGD	PGD	p:time	p:PGD	p:interaction
IL10	7.0 (3.8, 18.9)	9.7 (7.2, 25.4)	19.2 (4.0, 50.0)	15.9 (8.8, 32.1)	0.018	0.903	0.291
IL 6	51.8 (30.4, 124.7)	58.2 (21.3, 188.6)	78.5 (43.5, 209.0)	81.8 (44.8, 237.5)	0.003	0.773	0.314
IL 8	12.9 (4.9, 23.1)	14.1 (10.3, 24.2)	21.1 (10.6, 33.5)	23.2 (14.6, 45.1)	<0.001	0.226	0.555
TNF α	6.4 (4.8, 8.8)	8.8 (5.8, 12.3)	6.0 (4.4, 9.5)	8.7 (5.8, 12.0)	0.769	0.022	0.648

**TABLE 6:** Levels (pg/mL) of cytokines according to PGD0-2 vs. PGD3 at D0 and D1. Data expressed as median (Q1, Q3). *p*-values corresponding to non-parametric two-way mixed ANOVA.

	D0		D1		*p*-values		
	No PGD/PGD12	PGD3	No PGD/PGD12	PGD3	p:time	p:PGD3	p:interaction
IL10	8.8 (5.8, 23.2)	9.2 (6.8, 23.8)	20.6 (7.9, 40.7)	14.8 (6.4, 28.2)	0.001	0.419	0.040
IL 6	48.4 (20.9, 112.0)	164.3 (38.3, 302.2)	68.2 (35.7, 194.9)	125.8 (60.5, 443.6)	0.165	0.091	0.595
IL 8	13.0 (9.0, 22.7)	16.4 (10.2, 26.0)	19.9 (11.6, 34.3)	27.1 (17.1, 50.2)	<0.001	0.099	0.090
TNF α	7.0 (5.4, 10.5)	9.3 (6.0, 11.6)	7.4 (4.9, 11.8)	9.6 (6.4, 11.3)	0.182	0.618	0.615

**FIGURE 5:**
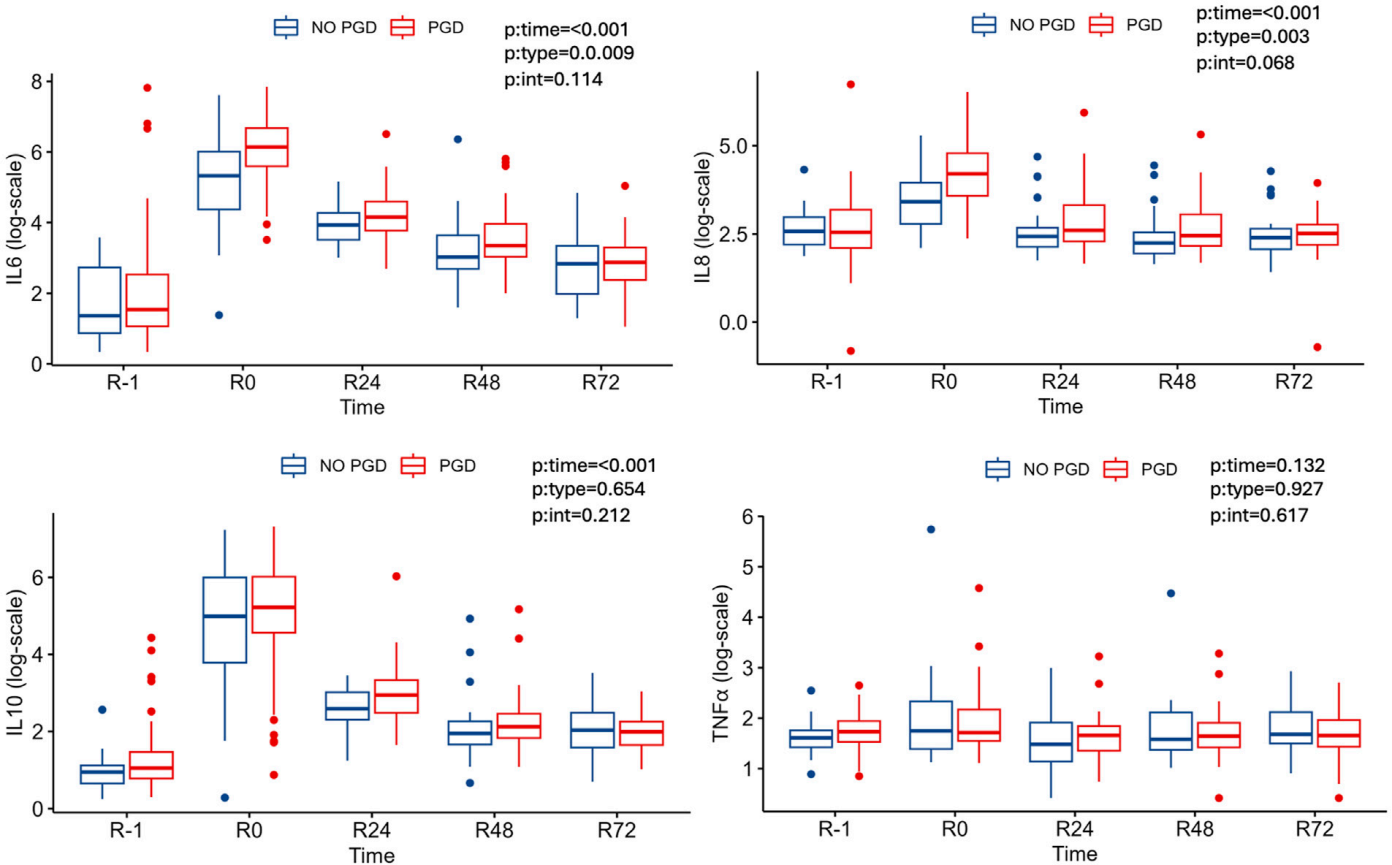
Cytokine and TNF-α median levels at R-1, R0, R24, R48, R72 according to PGD.

On the other hand, *recipients* experiencing PGD demonstrated elevated levels of IL-6 at R0 and a trend towards R48 and IL-8 at R0, R48, without differences between groups (Table 7; Figure 5). Furthermore, in the PGD grade 3 group, recipient plasma levels of IL-6 and IL-8 were significantly elevated at R0, R24 and R48 (Table 8).

**TABLE 7:** Levels (pg/mL) of cytokines according to PGD at R-1 to R72. Data expressed as median (Q1, Q3). *p*-values corresponding to non-parametric two-way mixed ANOVA.

	R-1		R0		R24		R48		R72		*p*-values		
	No PGD	PGD	No PGD	PGD	No PGD	PGD	No PGD	PGD	No PGD	PGD	p:time	p:PGD	p:interaction
IL10	2.6 (1.9, 3.1)	2.9 (2.2, 4.4)	146.5 (44.1, 402.0)	184.9 (95.7, 409.6)	13.3 (10.0, 20.5)	19.0 (12.0, 28.1)	7.0 (5.3, 9.6)	8.4 (6.2, 11.7)	7.6 (4.9, 12.1)	7.3 (5.2, 9.6)	<0.001	0.654	0.212
IL 6	3.9 (2.4, 15.3)	4.7 (2.9, 12.6)	205.2 (79.1, 407.5)	462.9 (269.4, 792.3)	51.0 (33.6, 71.9)	63.6 (43.5, 99.2)	20.7 (14.8, 38.1)	28.5 (20.7, 52.7)	17.1 (7.2, 28.2)	17.7 (10.8, 27.0)	<0.001	0.009	0.114
IL 8	13.1 (9.0, 19.6)	12.8 (8.2, 24.1)	30.2 (16.2, 52.1)	67.1 (35.9, 120.4)	11.4 (8.5, 14.5)	13.5 (9.9, 27.5)	9.4 (7.0, 12.7)	11.6 (8.7, 21.2)	11.0 (7.9, 14.1)	12.4 (9.0, 15.8)	<0.001	0.003	0.068
TNF α	5.0 (4.2, 5.8)	5.7 (4.6, 7.0)	5.8 (4.0, 10.3)	5.6 (4.7, 8.8)	4.4 (3.1, 6.8)	5.3 (3.9, 6.3)	4.9 (4.0, 8.3)	5.2 (4.2, 6.8)	5.4 (4.5, 8.3)	5.3 (4.2, 7.1)	0.132	0.927	0.617

**TABLE 8:** Levels (pg/mL) of cytokines according to PGD0-2 vs. PGD3 at R-1 to R72. Data expressed as median (Q1, Q3). *p*-values corresponding to non-parametric two-way mixed ANOVA.

	R-1		R0		R24		R48		R72		*p*-values		
	No PGD/PGD12	PGD3	No PGD/PGD12	PGD3	No PGD/PGD12	PGD3	No PGD/PGD12	PGD3	No PGD/PGD12	PGD3	p:time	p:PGD3	p:interaction
IL10	2.6 (2.0, 3.2)	2.7 (1.9, 4.3)	200.8 (44.9, 477.2)	160.6 (96.6, 290.4)	15.4 (10.0, 22.5)	22.0 (14.4, 33.2)	7.7 (5.7, 9.7)	9.2 (6.8, 18.8)	7.1 (4.9, 10.3)	7.7 (6.3, 10.4)	<0.001	0.576	0.321
IL 6	4.1 (2.7, 12.8)	5.2 (3.0, 13.3)	262.3 (101.1, 581.8)	441.0 (324.0, 1153.8)	53.3 (35.6, 78.0)	79.3 (44.7, 104.3)	25.3 (16.8, 55.2)	27.7 (23.6, 44.5)	17.3 (9.9, 27.8)	18.5 (9.6, 25.7)	<0.001	0.063	0.097
IL 8	12.2 (8.6, 19.2)	17.2 (8.3, 30.0)	42.3 (20.1, 88.9)	74.1 (48.1, 128.5)	11.8 (8.4, 16.1)	20.4 (11.8, 37.8)	10.1 (7.6, 14.4)	15.0 (10.6, 27.3)	11.2 (8.4, 13.8)	13.7 (8.5, 19.6)	<0.001	0.010	0.315
TNF α	5.4 (4.5, 6.7)	5.9 (5.1, 7.4)	5.5 (4.3, 9.2)	5.9 (4.7, 8.9)	4.6 (3.2, 6.5)	5.6 (4.3, 7.3)	5.0 (4.0, 7.1)	5.4 (4.2, 7.5)	5.2 (4.3, 7.6)	5.8 (4.1, 7.0)	0.270	0.374	0.834

### Mortality

Post-operative mortality rate was very low and similar in both groups without any additional deaths within 3 months after surgery (Table 4).

## Discussion

The main results of this prospective, controlled, multicenter, cohort-nested study are that: (1) DCD presents lower systemic inflammation than DBD in donors; (2) after LT, the time course of systemic, the incidence of PGD and mortality after LT is similar in BDB and DCD recipients; and (3)recipients from donors with elevated levels of tumor necrosis factor-alpha (TNF-α) have a higher incidence of PGD and PGD grade 3 with elevated levels of IL-6 and IL-8. These observations support the DCD as a viable LT option.

To our knowledge, this is the first prospective multicenter study comparing systemic inflammation, PGD and mortality in LT recipients according to DCD vs. DBD donors. Yet, previous experimental studies have shown the development of systemic inflammation following BD [4, 5, 9] Also, it is known that increased systemic inflammation may worsen ischemia-reperfusion-induced lung injury [9, 10], and has been associated with a higher incidence of PGD [11, 12]. Our results confirm that cytokine levels are increased after BD.

The first goal of this study was to investigate if the systemic inflammatory response elicited in DBD or DCD was different. We found that the plasma levels of IL-6, IL-8, IL-10, all well-established inflammatory markers [13] were higher in donors (D1) in DBD than in DCD. Of note, this occurred despite DBD had been treated with systemic corticosteroids more often. The role of treatment with corticosteroids in the management of DCD is a matter of debate [14] although it is widely used in practice. So far, only two experimental studies have indicated that it significantly reduces the plasma levels of several pro-inflammatory cytokines, warm ischemic injury [15] or myocardial edema [16]. However, these studies were conducted during *ex vivo* lung perfusion. Our results in real clinical practice suggest a small role for corticosteroid treatment in preventing systemic inflammation in lung DCD.

The second goal of our study was to compare the relationship of systemic inflammation with the incidence of PGD and short-term mortality in LT recipients from DBD and DCD donors. The relationship of PGD and systemic inflammation is a controversial issue since some previous studies have reported such a relationship [13, 17–20] whereas others did not [21]. We observed that recipients who presented PGD were transplanted from donors with elevated levels of TNF-α.

Finally, we found that lung reperfusion was followed by a rapid and similar increase in IL-6, IL-8 and IL-10 (not TNF-α) plasma levels in DBD and DCD recipients, followed by a reduction to normal levels in the next few hours, with a similar pattern in the two groups. These similarities likely explain why the incidence of PGD and mortality rate was not different in our study between DBD or DCD.

Our results may have clinical implications because they clearly show that DCD is not associated with increased systemic inflammation (as compared to DBD) and that the incidence of PGD and post-operative mortality seemed to be similar in LT recipients from DBD or DCD donors. This provides further support for the feasibility and safety of LT from DCD donors which, in turn, can stimulate DCD donation and contribute to alleviate the shortage of DBD donors and waiting lists.

The prospective, cohort-nested design of our study and the provision of the inflammatory status of donors before lung retrieval are strengths of our study. Among potential limitations we acknowledge that, although we estimated the needed sample size based on one of the most prominent cytokines investigated here (IL6), our results need to be replicated in other larger cohorts.

Brain death in humans is associated with higher levels of IL-6, IL-8 and IL-10, but this does not alter the biologic or clinical response of LT recipients. Yet, recipients transplanted from donors with higher TNF-α plasma levels (irrespective of DBD or DCD) have an increased incidence of PGD. These observations support the use of DCD in clinical practice.

## Data Availability

The raw data supporting the conclusion of this article will be made available by the authors, without undue reservation.
